# Supplementary material for: PD-1 blockade potentiates HIV latency reversal ex vivo in CD4+ T cells from ART-suppressed individuals
Source: Nat Commun. 2019 Feb 18;10:814. doi: 10.1038/s41467-019-08798-7 (PMC6379401; doi:10.1038/s41467-019-08798-7)
Supplement: Supplementary file 1 — Supplementary Information [file 41467_2019_8798_MOESM1_ESM.pdf]

Supplementary Figure 1

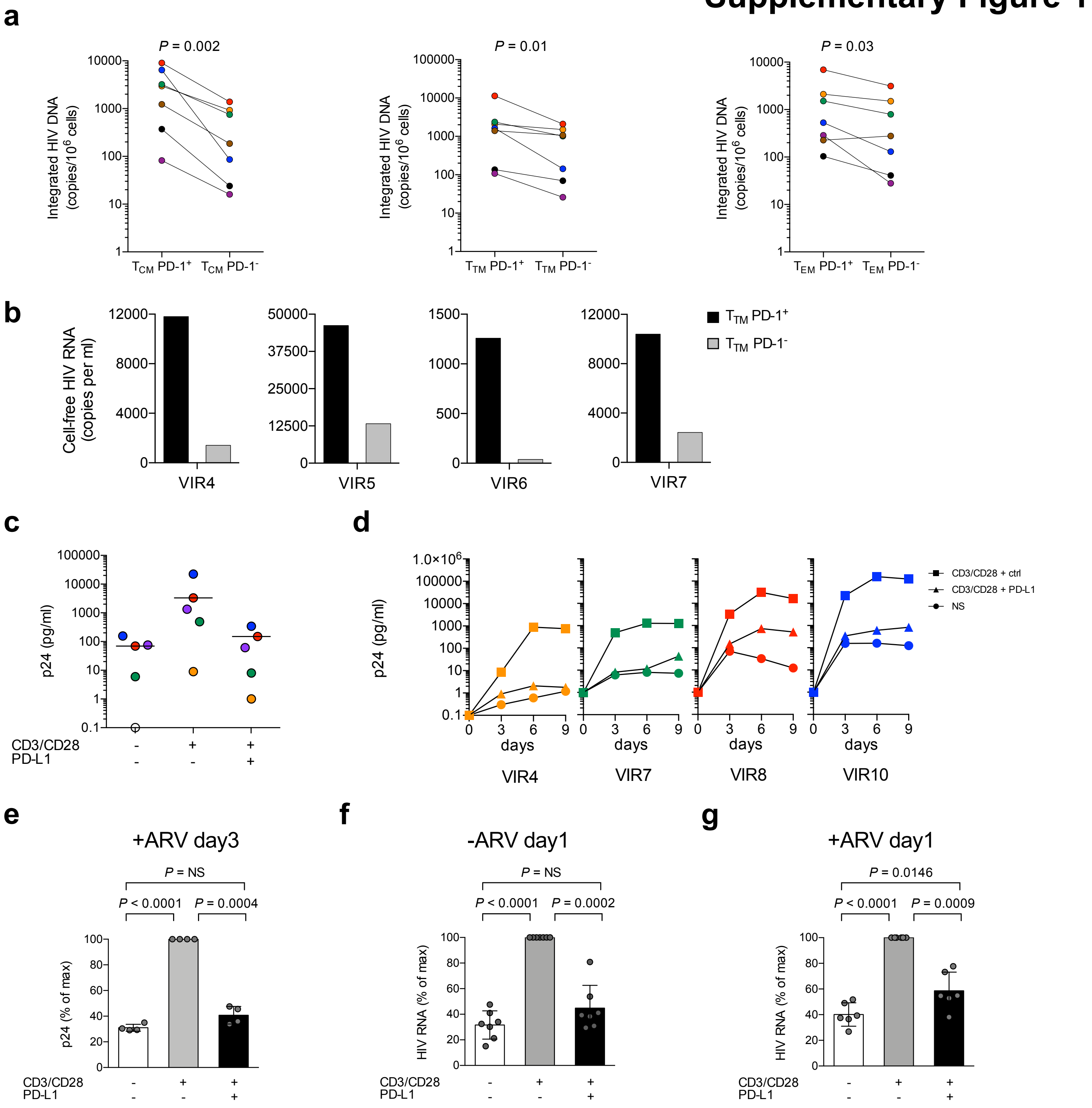

Supplementary figure 1: PD-1 is a marker of HIV-infected cells in viremic individuals and its engagement inhibits viral production. **(a)** Frequencies of cells harbouring integrated HIV DNA in T_{CM}, T_{TM} and T_{EM} CD4^{+} T-cell subsets sorted based on their expression of PD-1 (n=7 donors). P values were obtained from paired t-test analysis of the log transformed values. **(b)** Viral production measured by RT-PCR in 1-day culture supernatants of T_{TM} CD4^{+} T cells from viremic individuals sorted based on their expression of PD-1 (n=4 donors). **(c)** Viral production measured by p24 release in 5 independent donors. Combined analysis of the 5 donors using normalized values is shown in Fig. 1a. **(d)** Viral production measured by p24 release in 4 additional donors as in Fig. 1b. Combined analysis of the 5 donors using normalized values is shown in Fig. 1c. **(e)** Relative viral production measured by p24 ELISA in 3-days culture supernatants of CD4^{+} T cells isolated from viremic individuals and stimulated in the presence or absence of PD-L1 in the presence of ARVs (means and standard deviations from n=4 donors). P values were obtained from paired t-test analysis. **(f)** Same as in (e) with viral production measured by RT-PCR after 1-day of culture in the absence of ARVs (means and standard deviations from n=7 donors). P values were obtained from paired t-test analysis. **(g)** Same as in (e) with viral production measured after 1-day of culture in the presence of ARVs (means and standard deviations from n=6 donors). P values were obtained from paired t-test analysis.

Supplementary Figure 2

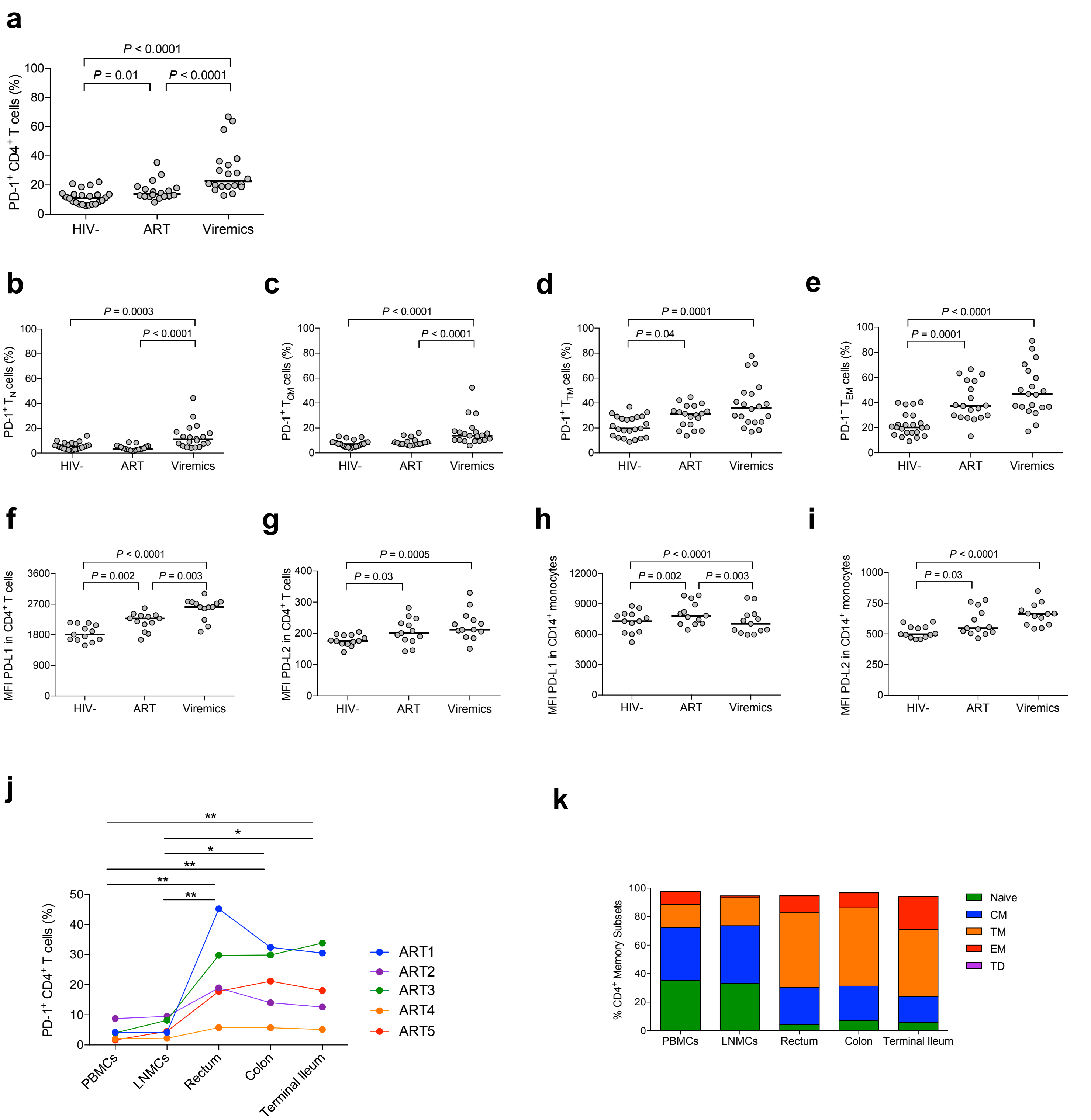

Supplementary figure 2: PD-1, PD-L1 and PD-L2 expressions are not normalized by ART and PD-1 expression is elevated in tissues enriched in differentiated CD4+ T cells. **(a)** Frequencies of PD-1 expressing CD4^{+} T cells from HIV-negative controls, ART suppressed and viremics HIV-infected individuals (n=23, n=19 and n=20, respectively). **(b)**, **(c)**, **(d)** and **(e)** Frequencies of PD-1 expressing Naïve, T_{CM}, T_{TM} and T_{EM} CD4^{+} T cells from the same individuals. **(f)(g)** Mean fluorescence intensity (MFI) of PD-L1 and PD-L2 in CD4^{+} T cells from HIV-negative controls, ART suppressed and viremics HIV-infected individuals (n=13 in each group). **(h)(i)** MFI of PD-L1 and PD-L2 in CD14^{+} cells from the same individuals. P values were obtained from Mann-Whitney U test analysis. **(j)(k)** Frequencies of PD-1 expressing CD4^{+} T cells and memory CD4^{+} T cell subsets (respectively) from peripheral blood (PBMCs), and matched lymph node (LNMCs), rectum, colon and terminal ileum from 5 virally suppressed individuals. P values were obtained from 2-way ANOVA analysis (**: p<0.01; *: p<0.05).

Supplementary Figure 3

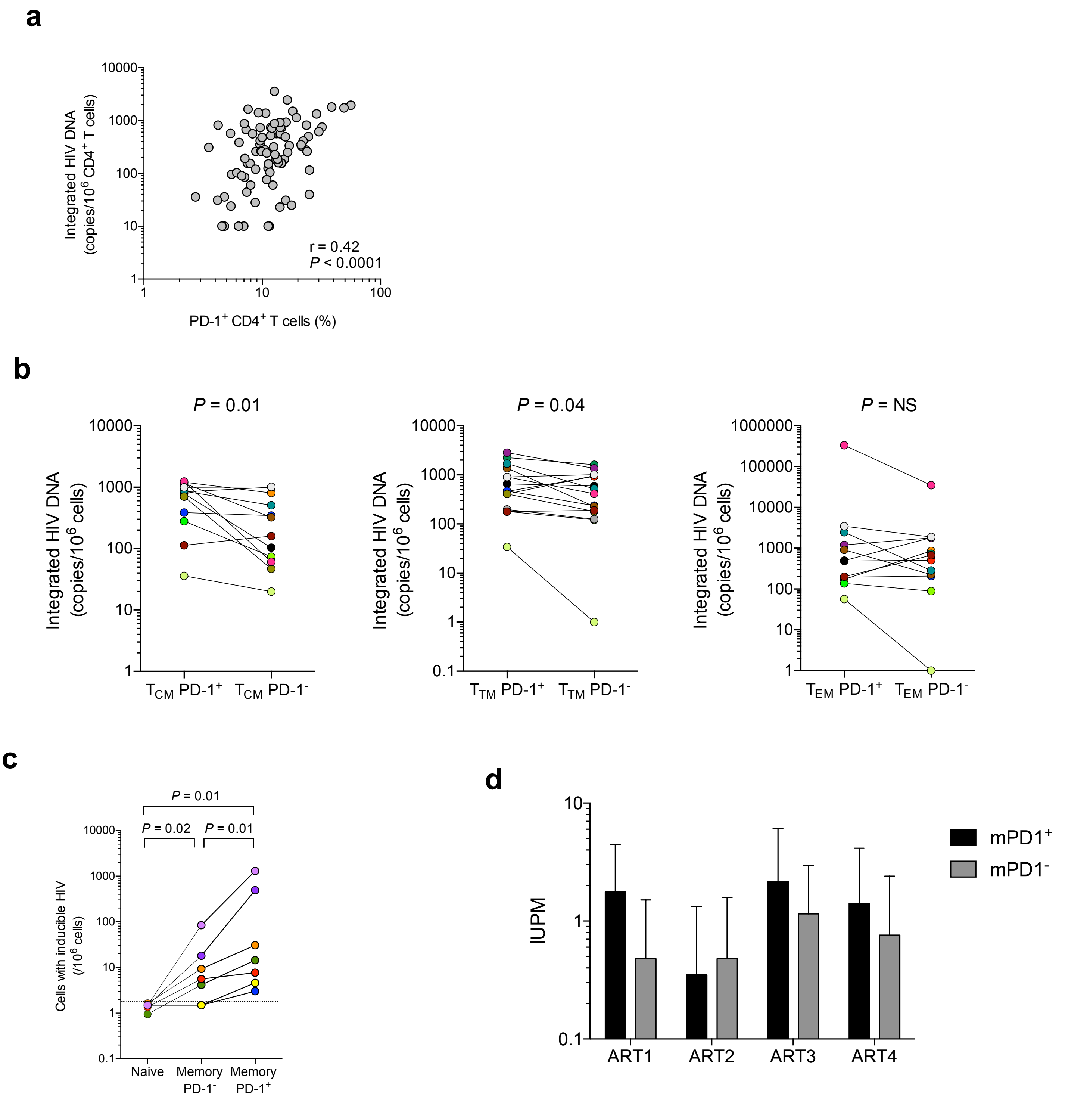

Supplementary figure 3: PD-1 is a marker of HIV-infected cells in virally suppressed individuals on ART. **(a)** Association between the frequency of CD4$^+$ T cells harbouring integrated HIV DNA and the frequency of CD4$^+$ T cells expressing PD-1 (n=89). P and r values were obtained from Pearson analysis. **(b)** Frequencies of cells harbouring integrated HIV DNA in T_{CM}, T_{TM} and T_{EM} CD4$^+$ T-cell subsets sorted based on their expression of PD-1 (n=12, n=16 and n=13, respectively). P values were obtained from paired t-test analysis of the log transformed value. **(c)** Frequency of cells harbouring inducible msRNA measured by TILDA in memory CD4$^+$ T cells sorted based on their expression of PD-1 (n=7). P values were obtained from paired t-test analysis of the log transformed value. **(d)** Frequency of cells harbouring replication-competent HIV measured by QVOA in memory CD4$^+$ T cells sorted based on their expression of PD-1 (n=4). Results are expressed as infectious units per million cells (IUPM). Error bars represent 95% confidence intervals.

Supplementary Figure 4

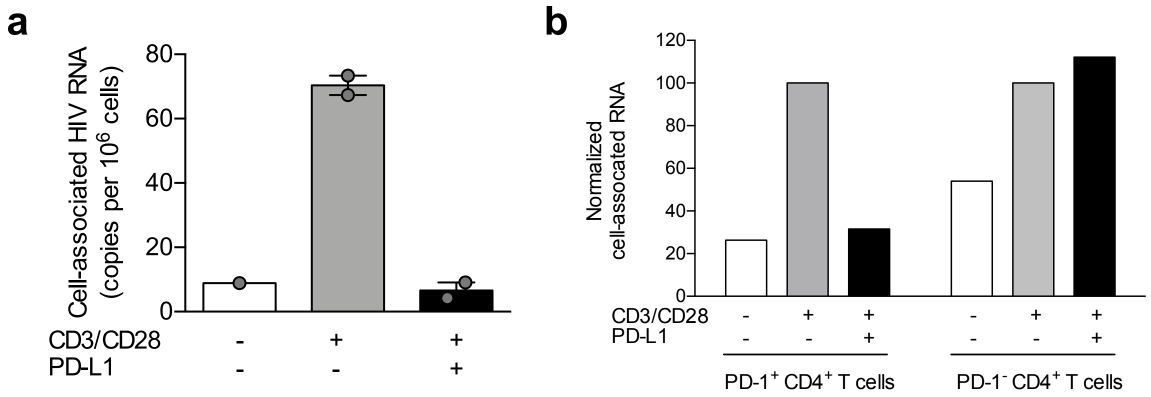

Supplementary figure 4: PD-1 engagement inhibits viral reactivation from latently infected cells. (a) Cell-associated HIV RNA measured by RT-PCR in CD4+ T cells from a virally suppressed individual after 24h of stimulation in the presence or absence of PD-L1 and in the presence of ARVs (means and standard deviations from duplicate measures). **(b)** Cell-associated HIV RNA measured by RT-PCR in memory CD4+ T cells sorted based on their expression of PD-1 from a virally suppressed individual after 24h of stimulation in the presence or absence of PD-L1 and in the presence of ARVs.

Supplementary Figure 5

a

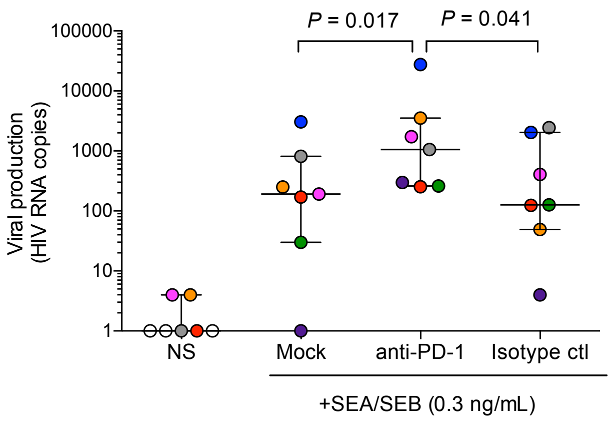

b

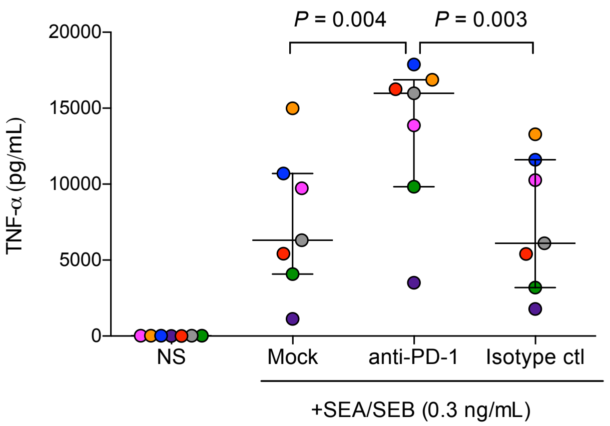

c

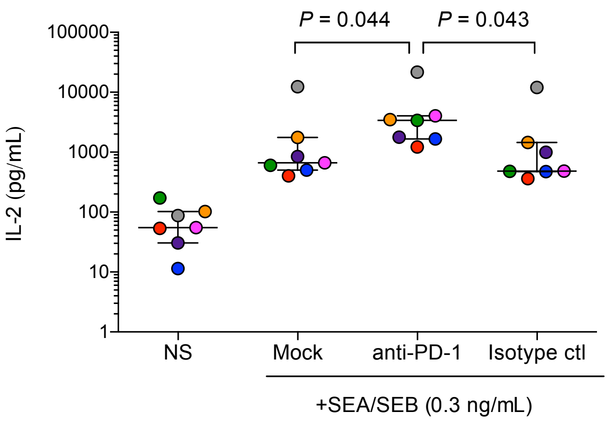

Supplementary figure 5: PD-1 blockade enhances TCR-induced viral production. **(a)** Viral production measured by RT-PCR in 3-days culture supernatants of CD8-depleted PBMCs isolated from virally suppressed individuals and stimulated with SEA/SEB (0.3ng/mL) in the presence of pembrolizumab or the appropriate isotype control (10µg/mL) (median and interquartile range from n=7 donors). P values were obtained from paired t-test analysis of the log transformed values +1. **(b)(c)** TNF-α and IL-2 productions measured by multi-array technology in cell culture supernatants from the experiment described in (a) (median and interquartile range from n=7 donors). P values were obtained from paired t-test analysis.

Supplementary Figure 6

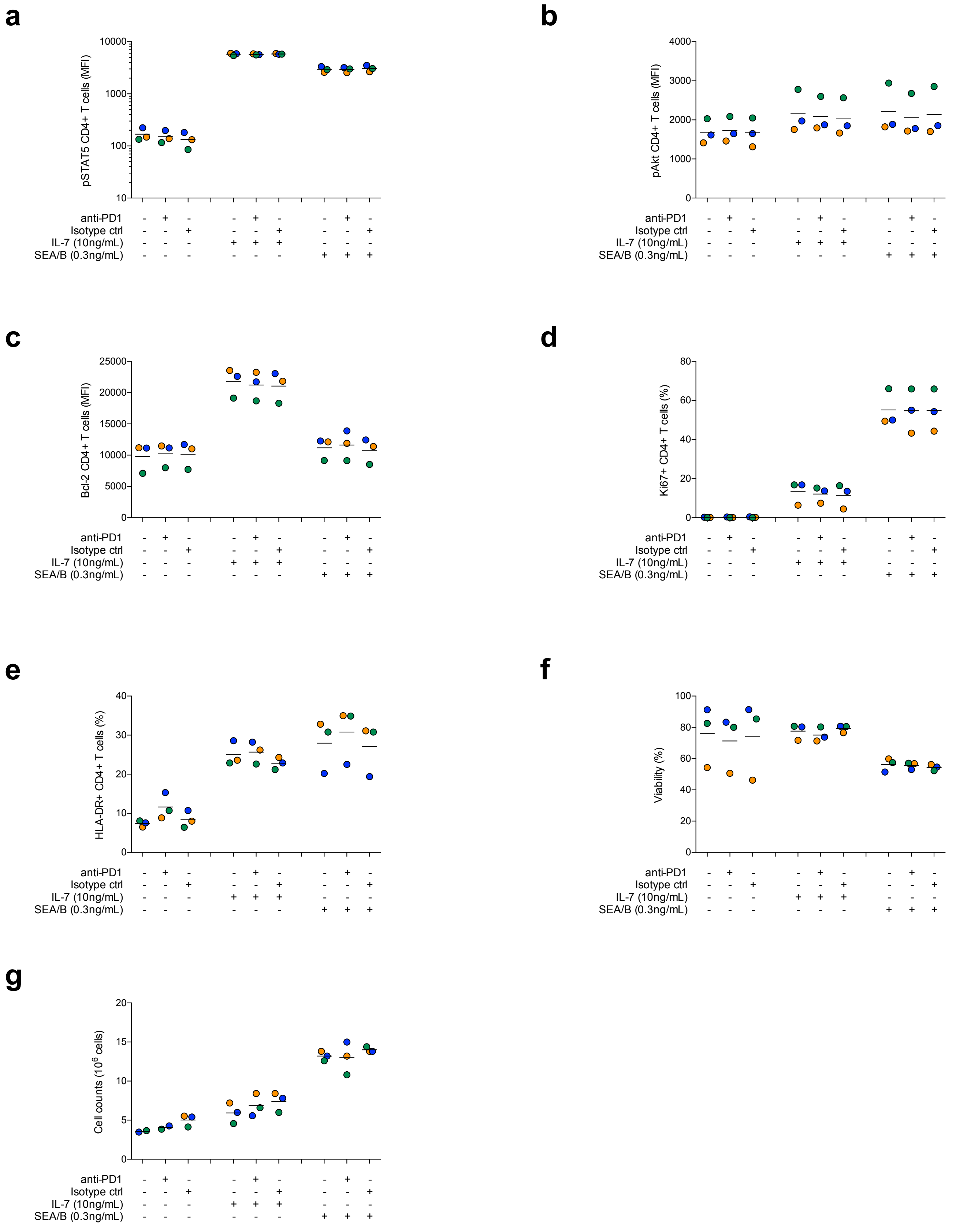

Supplementary figure 6: *Ex vivo* PD-1 blockade does not affect markers of T cell homeostasis, proliferation, survival and activation. **(a-e)** PBMCs isolated from virally suppressed individuals were cultured for 8 days in the presence or absence of IL-7 (10ng/mL) or SEA/SEB (0.3ng/mL) in the presence of pembrolizumab or isotype control (10μg/mL). Level of STAT5 and AKT phosphorylation (18h and 48h, respectively), Bcl-2 (120h), Ki67 (120h), HLA-DR (120h) were measured by flow cytometry (a-e, respectively). Viability and cell counts were assessed at the end of the culture (192h) (f and g, respectively).

Supplementary Figure 7

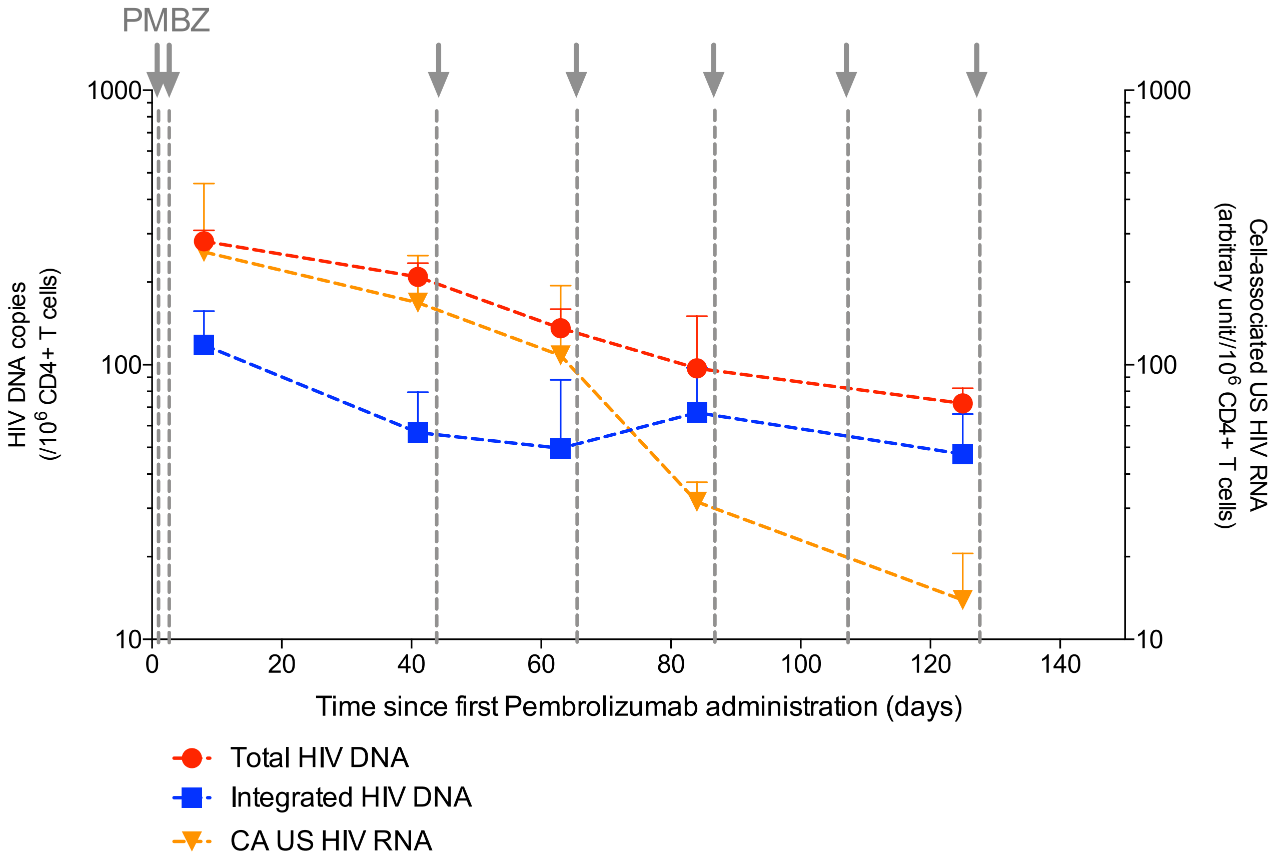

Supplementary figure 7: PD-1 blockade reduces markers of HIV persistence *in vivo*. Markers of HIV persistence (frequency of $CD4^+$ T cells harbouring total and integrated HIV DNA (red circles and blue squares, respectively), cell-associated HIV RNA (orange triangles) during pembrolizumab therapy (7 infusions, grey arrow) in a virally suppressed HIV-infected individual with metastatic malignant melanoma.

Supplementary Figure 8

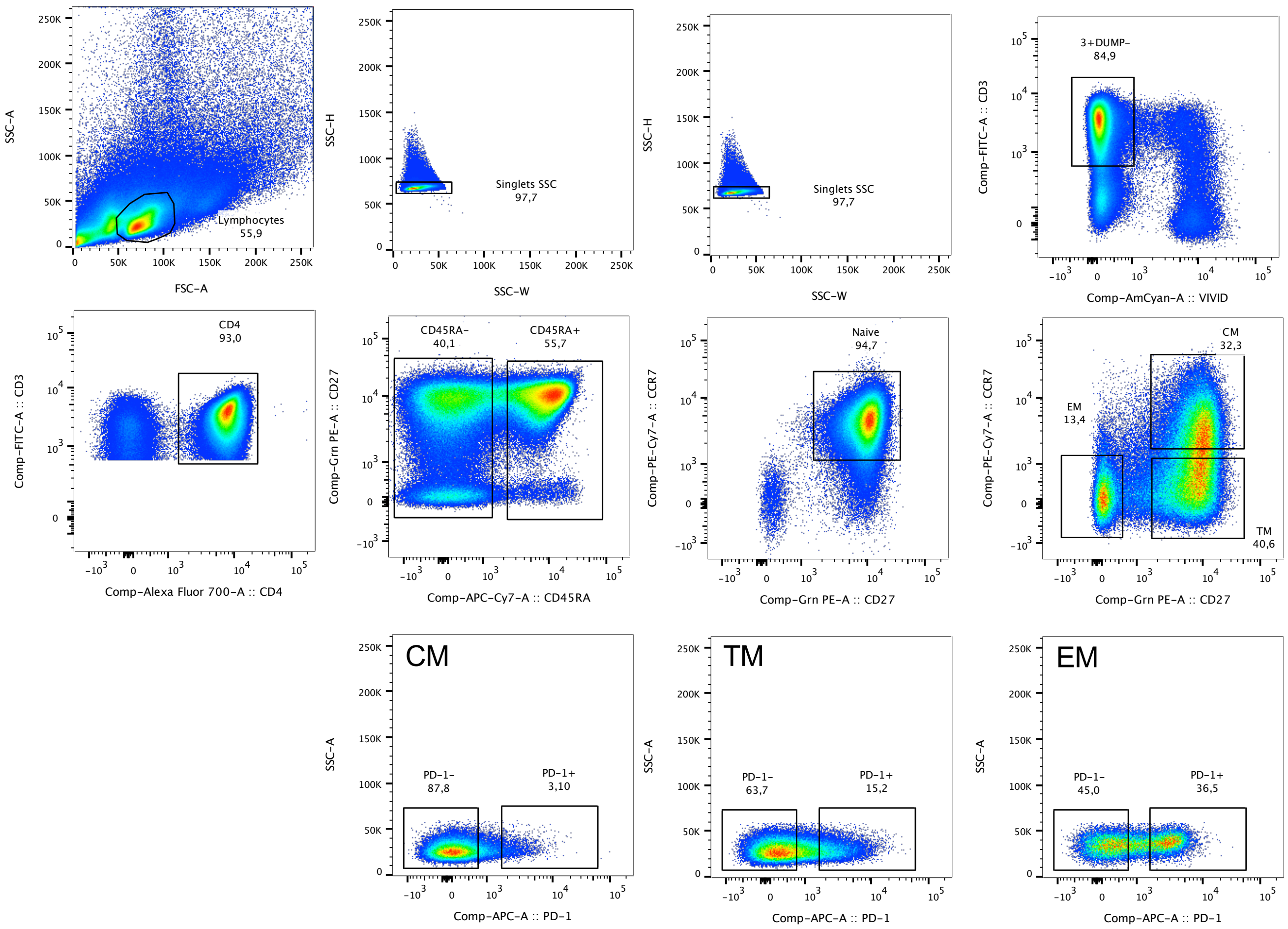

Supplementary figure 8: Gating strategy. Representative dot plots of the gating strategy for the isolation of PD-1^{+} and PD-1^{-} T_{CM}, T_{TM} and T_{EM} CD4^{+} cells.

Supplementary Table 1

Test	Primer/probe	Sequence
Integrated HIV DNA	HCD3 out 5'	5'-ACT GAC ATG GAA CAG GGG AAG-3'
	HCD3 out 3'	5'-CCA GCT CTG AAG TAG GGA ACA TAT-3'
	HCD3 in 5’	5'-GGC TAT CAT TCT TCT TCA AGG T-3'
	HCD3 in 3'	5'-CCT CTC TTC AGC CAT TTA AGT A-3'
	CD3 FamZen	5'-/56-FAM/AG CAG AGA A/ZEN/C AGT TAA GAG CCT CCA T/3IABkFQ/-3'
	ULF1	5'-ATG CCA CGT AAG CGA AAC TCT GGG TCT CTC TDG TTA G AC-3'
	Alu1	5'-TCC CAG CTA CTG GGG AGG CTG AGG-3'
	Alu2	5'-GCC TCC CAA AGT GCT GGG ATT ACA G-3'
	LambdaT	5'-ATG CCA CGT AAG CGA AAC T -3'
	UR2	5'- CTG AGG GAT CTC TAG TTA CC-3'
	UHIV FamZen	5'-/56-FAM/CA CTC AAG G/ZEN/C AAG CTT TAT TGA GGC /3IABkFQ/-3'
TILDA	tat1.4	5’-TGG CAG GAA GAA GCG GAG A-3’
	rev	5’-GGA TCT GTC TCT GTC TCT CTC TCC ACC-3’
	tat2	5’-ACA GTC AGA CTC ATC AAG TTT CTC TAT CAA AGC A-3’
	MS-HIV-FamZen	5’-/56-FAM/TTC CTT CGG/ZEN/GCC TGT CGG GTC CC/3IABkFQ/-3’
HIV RNA	ULF1	5'-ATG CCA CGT AAG CGA AAC TCT GGG TCT CTC TDG TTA G AC-3'
	UR1	5’- CCA TCT CTC TCC TTC TAG C -3'
	LambdaT	5'-ATG CCA CGT AAG CGA AAC T -3'
	UR2	5'- CTG AGG GAT CTC TAG TTA CC-3'
	UHIV FamZen	5'-/56-FAM/CA CTC AAG G/ZEN/C AAG CTT TAT TGA GGC /3IABkFQ/-3'

Supplementary table 1: Primers and probes sequences.
