# Supplementary material for: PD-1 blockade potentiates HIV latency reversal ex vivo in CD4+ T cells from ART-suppressed individuals
Source: Nat Commun. 2019 Feb 18;10:814. doi: 10.1038/s41467-019-08798-7 (PMC6379401; doi:10.1038/s41467-019-08798-7)
Supplement: Supplementary file 3 — Reporting Summary [file 41467_2019_8798_MOESM3_ESM.pdf]

nature research

Corresponding author(s): *Double-blind peer review submissions: write DBPR and your manuscript number here instead of author names.*

Reporting Summary

Nature Research wishes to improve the reproducibility of the work that we publish. This form provides structure for consistency and transparency in reporting. For further information on Nature Research policies, see Authors & Referees and the Editorial Policy Checklist.

Statistical parameters

When statistical analyses are reported, confirm that the following items are present in the relevant location (e.g. figure legend, table legend, main text, or Methods section).

n/a	Confirmed	
☐	☒	The exact sample size (*n*) for each experimental group/condition, given as a discrete number and unit of measurement
☐	☒	An indication of whether measurements were taken from distinct samples or whether the same sample was measured repeatedly
☐	☒	The statistical test(s) used AND whether they are one- or two-sided<br>*Only common tests should be described solely by name; describe more complex techniques in the Methods section.*
☐	☒	A description of all covariates tested
☐	☒	A description of any assumptions or corrections, such as tests of normality and adjustment for multiple comparisons
☐	☒	A full description of the statistics including central tendency (e.g. means) or other basic estimates (e.g. regression coefficient) AND variation (e.g. standard deviation) or associated estimates of uncertainty (e.g. confidence intervals)
☐	☒	For null hypothesis testing, the test statistic (e.g. *F*, *t*, *r*) with confidence intervals, effect sizes, degrees of freedom and *P* value noted<br>*Give P values as exact values whenever suitable.*
☒	☐	For Bayesian analysis, information on the choice of priors and Markov chain Monte Carlo settings
☒	☐	For hierarchical and complex designs, identification of the appropriate level for tests and full reporting of outcomes
☒	☐	Estimates of effect sizes (e.g. Cohen's *d*, Pearson's *r*), indicating how they were calculated
☐	☒	Clearly defined error bars<br>*State explicitly what error bars represent (e.g. SD, SE, CI)*

Our web collection on statistics for biologists may be useful.

Software and code

Policy information about availability of computer code

Data collection	BD FACSDiva software, RotorGeneQ software (Qiagen), QuantStudio software v3.1 (Applied Biosystems)
Data analysis	FlowJo v9, Prism 6.0 (GraphPad Software)

For manuscripts utilizing custom algorithms or software that are central to the research but not yet described in published literature, software must be made available to editors/reviewers upon request. We strongly encourage code deposition in a community repository (e.g. GitHub). See the Nature Research guidelines for submitting code & software for further information.

Data

Policy information about availability of data

All manuscripts must include a data availability statement. This statement should provide the following information, where applicable:

- Accession codes, unique identifiers, or web links for publicly available datasets
- A list of figures that have associated raw data
- A description of any restrictions on data availability

All data are available from the corresponding author upon reasonable request.

Field-specific reporting

Please select the best fit for your research. If you are not sure, read the appropriate sections before making your selection.

☒ Life sciences ☐ Behavioural & social sciences ☐ Ecological, evolutionary & environmental sciences

For a reference copy of the document with all sections, see nature.com/authors/policies/ReportingSummary-flat.pdf

Life sciences study design

All studies must disclose on these points even when the disclosure is negative.

Sample size	Twenty HIV-infected viremic participants, 120 HIV-infected virally suppressed ART-treated individuals and 23 HIV negative people were recruited in the study.
Data exclusions	As mentioned in the Method section, for the experiments aimed at evaluating the latency reversal ability of PD-1 blockade, samples with spontaneous viral production (>10 HIV RNA copies in the non-stimulated condition) and/or inconsistent replicate values attributed to low frequency of reservoir cells were excluded.
Replication	All data acquired are reported, therefore the reproducibility can be judged within the manuscript.
Randomization	No randomization was performed.
Blinding	Blinding was not performed.

Reporting for specific materials, systems and methods

Materials & experimental systems

n/a	Involved in the study	
☐	☒	Unique biological materials
☐	☒	Antibodies
☒	☐	Eukaryotic cell lines
☒	☐	Palaeontology
☒	☐	Animals and other organisms
☐	☒	Human research participants

Methods

n/a	Involved in the study	
☒	☐	ChIP-seq
☐	☒	Flow cytometry
☒	☐	MRI-based neuroimaging

Unique biological materials

Policy information about availability of materials

Obtaining unique materials	PD-L1 chimeric protein was provided by Gordon Freeman.<br>PD-1 blockade antibody, Pembrolizumab, was provided by Merck.<br>pCDK9 (pS175) was provided by Merck.

Antibodies

Antibodies used	CD3-Alexa700 (clone UCHT1, BD#557943)<br>CD3-PB (clone UCHT1, BD#558117)<br>CD4-QDot605 (clone S3.5, Invitrogen#Q10008)<br>CD4-Alexa700 (clone RPA-T4, BD#557922)<br>CD4-BUV496 (clone SK3, BD#564651)<br>CD4-PB (clone RPA-T4, BD#558116)<br>CD8-PB (clone RPA-T8, BD#558207)<br>CD8-PerCPCy5.5 (clone RPA-T8, BD#560662)<br>CD8-BUV395 (clone RPA-T8, BD#563795)<br>CD14-V500 (clone M5E2, BD#561391)<br>CD14-FITC (clone M5E2, BD#555397)<br>CD19-AmCyan (clone SJ25C1, BD#339190)<br>LIVE/DEAD Aqua marker (Invitrogen#L34957)<br>CD45RA-APC-H7 (clone HI100, BD#560674)

	CD27-BV650 (clone O323, Biolegend#302828)<br>CCR7-PE-Cy7 (clone 3D12, BD#557648)<br>PD-1-APC (clone MIH4, eBioscience#17-9969-42)<br>PD-1-BUV737 (clone EH12.1, BD#565299)<br>PD-L1-PE-Cy7 (clone MIH1, BD#558017)<br>PD-L2-PE (clone MIH18, BD#558066)<br>CD38-PE (clone HIT2, BD#555460)<br>HLA-DR-BUV395 (clone G46-6, BD#564040)<br>HLA-DR-APC (clone G46-6, BD#559866)<br>CD69-APC (clone FN50, BD#555533)<br>CD25-BUV737 (clone 2A3, BD#564385)<br>Ki67-FITC (BD#556026)<br>Bcl-2-V450 (clone Bcl-2/100, BD#560637)<br>pSTAT5-AF647 (pY694, BD#612599)<br>pAkt-PE-CF594 (pS473, BD#562465)<br>CyclinT1-TRIC (SantaCruz#8127)
Validation	All antibodies are commercially available but the anti-human pCDK9 (provided by Merck). We performed titrations to determine optimal antibody concentrations.

Human research participants

Policy information about studies involving human research participants

Population characteristics	Untreated, viremic participants were defined as HIV-infected individuals who were either treatment naïve, or who had been off antiviral medication for over 6 months. ART treated, virally suppressed participants were receiving ART for >3 years and HIV RNA <40 copies/mL for at least 3 years. HIV negative participants were individuals who were seronegative for HIV. No other selection was performed.
Recruitment	All participants signed inform consent approved by the Martin Memorial Health Systems (FL, USA), the UCSF (CA, USA), the Royal Victoria Hospital and the CHUM hospital (QC, Canada) review boards (IRB #10-1320, Ref # 068192 and FWA #00004139, respectively).

Flow Cytometry

Plots

Confirm that:

☒ The axis labels state the marker and fluorochrome used (e.g. CD4-FITC).

☒ The axis scales are clearly visible. Include numbers along axes only for bottom left plot of group (a 'group' is an analysis of identical markers).

☒ All plots are contour plots with outliers or pseudocolor plots.

☒ A numerical value for number of cells or percentage (with statistics) is provided.

Methodology

Sample preparation	PBMCs were isolated from leukapheresis product by Ficoll density gradient centrifugation. Single cell suspension from lymph node and gut biopsies were obtained after mechanical and enzymatic digestion, respectively.
Instrument	Flow cytometry acquisition was done with BD LSR II flow cytometer (BD), and sorted cells were collected using a FACSAria II cell sorter (BD Biosciences).
Software	Acquisition was performed with BD FACSDiva software and analysis was performed with FlowJo V9.
Cell population abundance	Sorted subsets were separated by FACSAria to very high purity (98.0%) as measured by flow cytometry. Cell population abundance varied between subjects and are reported in the manuscript when relevant.
Gating strategy	A supplementary Figure will be added to the manuscript to describe all the relevant gating strategies.

☒ Tick this box to confirm that a figure exemplifying the gating strategy is provided in the Supplementary Information.
